# Developing a model for estimating the activity of colonic microbes after intestinal surgeries

**DOI:** 10.1371/journal.pone.0253542

**Published:** 2021-07-28

**Authors:** Andrew Marcus, Taylor L. Davis, Bruce E. Rittmann, John K. DiBaise, Elvis A. Carnero, Karen Corbin, Steven R. Smith, Rosa Krajmalnik-Brown

**Affiliations:** 1 Biodesign Swette Center for Environmental Biotechnology, Arizona State University, Tempe, AZ, United States of America; 2 Biodesign Center for Health Through Microbiomes, Arizona State University, Tempe, AZ, United States of America; 3 Division of Gastroenterology, Mayo Clinic, Scottsdale, AZ, United States of America; 4 Translational Research Institute, AdventHealth, Orlando, FL, United States of America; Washington State University - Spokane, UNITED STATES

## Abstract

**Background:**

The large intestine provides a compensatory role in energy recovery when surgical interventions such as extensive small intestinal resections or bypass operations lower the efficiency of nutrient absorption in the upper gastrointestinal (GI) tract. While microorganisms in the colon are known to play vital roles in recovering energy, their contributions remain to be qualified and quantified in the small intestine resection.

**Objective:**

We develop a mathematical model that links nutrient absorption in the upper and lower GI tract in two steps.

**Methods:**

First, we describe the effects of small intestine resection on the ileocecal output (ICO), which enters the colon and provides food for microbes. Second, we describe energy recovered by the colon’s microorganisms via short-chain fatty acid (SCFA) production. We obtain model parameters by performing a least-squares regression analysis on clinical data for subjects with normal physiology and those who had undergone small intestine resection.

**Results:**

For subjects with their intestines intact, our model provided a metabolizable energy value that aligns well with the traditional Atwater coefficients. With removal of the small intestine, physiological absorption became less efficient, and the metabolizable energy decreased. In parallel, the inefficiencies in physiological absorption by the small intestine are partly compensated by production of short-chain fatty acids (SCFA) from proteins and carbohydrates by microorganisms in the colon. The colon recovered more than half of the gross energy intake when the entire small intestine was removed. Meanwhile, the quality of energy absorbed changed, because microbe-derived SCFAs, not the original components of food, become the dominant form of absorbed energy.

**Conclusion:**

The mathematical model developed here provides an important framework for describing the effect of clinical interventions on the colon’s microorganisms.

## 1. Introduction

Microorganisms in the large intestine influence multiple facets of human health, including the human’s metabolism, immune system, and gut-brain communication [1–3]. Intestinal microorganisms convert nutrients that escaped digestion in the upper gastrointestinal (GI) tract, secretions of the upper GI tract, and sloughed epithelial cells into small organic compounds that the large intestine can readily absorb. Short-chain fatty acids (SCFAs)—particularly acetate, propionate, and butyrate—are the primary products of anaerobic microbial metabolism. In healthy individuals with normal physiology, SCFAs contribute 5 to 10% of energy uptake [4, 5]. Dietary and surgical interventions can alter the structure of the microbial community [6–10], and microorganisms in turn can mediate the effects of these interventions on the human host [3, 11].

Broadly speaking, the human GI tract is divided into the upper and lower GI tracts. The upper GI tract extends from the oral cavity to the ileocecal valve. The lower GI tract is also called the colon. Digestion within the small intestine occurs mostly in the jejunum and ileum via a combination of mechanical action and enzymatic reactions [12]. Microbial metabolism in the upper GI tract is of minor importance, and the density of microorganisms goes from about 10^1^ to 10^3^ mg/ml in the stomach to about 10^7^ to 10^9^ mg/ml in the ileum [13]. While the proximal regions of the upper GI tract are aerobic, its terminal regions are hypoxic. The large intestine is where microorganisms become dominant. The density of microorganisms in the lumen of the large intestine is about 10^10^ to 10^12^ mg/ml, and the environment is fully anoxic [14].

While the small intestine is the primary location for energy absorption, it has been reported that the large intestine becomes more important in energy absorption capability for individuals who have had significant small intestinal resections [15]. Although it is logical that microbial activity in the large intestine becomes responsible for more energy uptake in subjects with a shortened small intestine, the role of the microorganisms is poorly understood. Here, we develop and use a mathematical model to interpret the role of the microbial community in energy uptake for the subjects in Norgaard et al. [15].

In the human gut, the contents are transported from proximal to distal regions. This means that processes in the upper intestine have effects on the microbiological community of the colon. Of paramount importance is the quantity and quality of the organic substrate (food to the microorganisms) that enters the colon. Food available to the colonic microorganisms is what passes through the ileocecal valve in what is called the *ileocecal output*. Directly measuring the ileocecal output is practically impossible when the entire gastrointestinal system is intact. However, the contents of ileocecal output are readily available for individuals who have undergone removal of the entire colon [16] and creation of an ileostomy.

To date, mathematical modeling of the interactions of intestinal microorganisms and the human host has been focused mainly on the large intestine, since this is where the vast majority of the microorganisms reside [17–20]. One challenge for modeling microbial processes in the large intestine is having a good estimate of the quantity and quality of substrates entering the large intestine. Accurately representing the ileocecal output makes it possible for a mathematical model to provide a good estimate of microbiological contributions to human energy metabolism for individuals with an intact or resected small intestine.

Here, we develop a stoichiometry-based method for estimating the extent of dietary substrate absorption for individuals with an intact small intestine and others who have undergone intestinal resection. First, we examine the literature to estimate the extent of the dietary absorption in the upper GI tract and use this information to estimate the quantity and quality of the *ileocecal output*. Then, we estimate the amount of SCFAs and biomass produced by microbial metabolism in the large intestine. We also track iso-butyrate and aromatic compounds, which are useful indicators of protein biodegradation. Finally, we discuss the consequences of an intestinal resection on microbial activity and human energy uptake.

## 2. Model formulation

We develop a multicomponent model that tracks 14 components: available starch, sugars, resistant starch (RS), non-starch polysaccharides (NSP), proteins, amino acids, fats, acetic acid, propionic acid, n-butyric acid, iso-butyric acid, aromatics, carbohydrate-utilizing bacteria, and protein-utilizing bacteria. We refer to the sum of available starch and sugars as AvSS. The sum of AvSS, NSP, and RS constitutes carbohydrates. In the context of human nutrition and diet, carbohydrates, fats, and proteins are collectively referred to as macronutrients.

**Fig 1:**
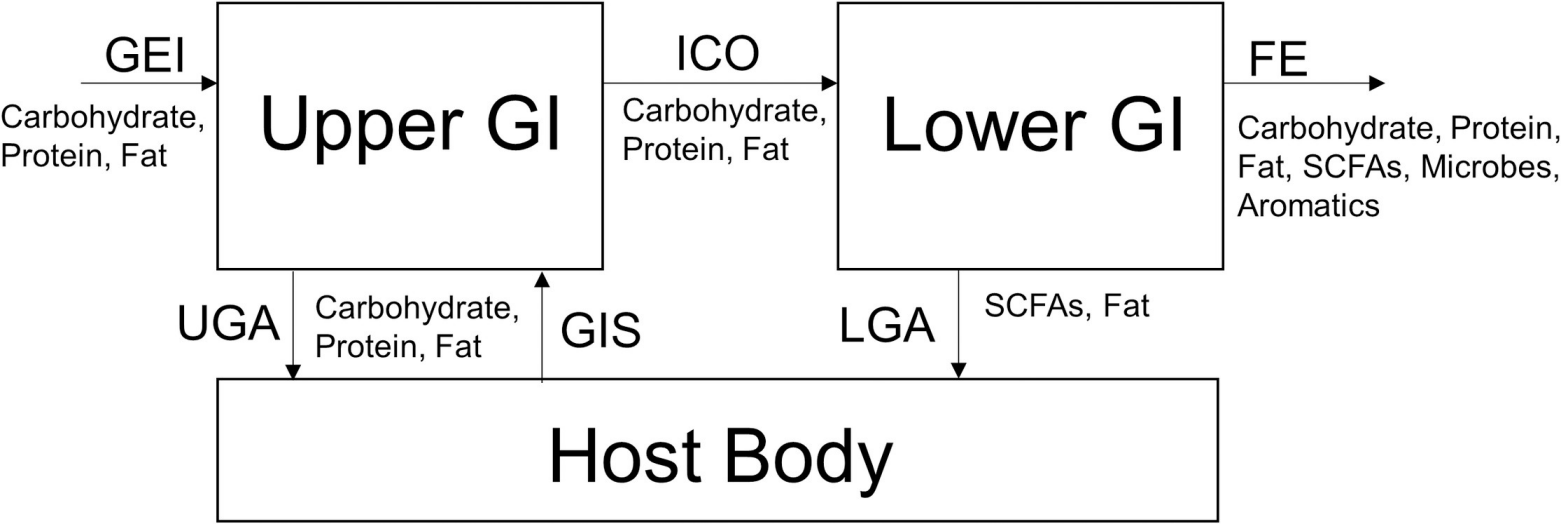
The overall material flows of our model. The abbreviations are gross energy intake (GEI), upper GI absorption (UGA), gastrointestinal secretions (GIS), ileocecal output (ICO), lower GI absorption (LGA), and fecal energy (FE). While not explicitly modeled, the gastrointestinal host acts as the source for GIS and sink for UGA and LGA.

Fig 1 illustrates the overall material flow in our model. We represent the human digestive tract using two compartments, the upper and lower GI tracts. The upper GI tract receives Gross Energy Intake (GEI) from the diet as carbohydrates, proteins, and fats and gastrointestinal secretions (GIS) from the body. The small intestine absorbs a fraction of these components via upper GI absorption (UGA). The remaining fraction leaves the small intestine as ileocecal output (ICO) and enters the colon to serve as substrates for microorganisms. In the colon, microorganisms convert carbohydrates to SCFAs and proteins to branched chain fatty acids and aromatics. The colon absorbs SCFAs and fat, (termed here as lower GI absorption (LGA)) and excretes all remaining materials as fecal energy (FE) [5]. The following sections describe how our model describes all model components, physiological processes in the upper GI, and microbiological and physiological processes in the colon.

### 2.1 Units conventions

The literature for human nutrition and metabolism represents the macronutrients based on their caloric values and weight. The meaning of caloric values depends on the physiological context. Three types of caloric values are GEI, Digestible Energy Intake (DEI), and Metabolizable Energy Intake (MEI). GEI is the heat value of food determined via calorimetry when food is completely oxidized with oxygen. In practice, food scientists estimate GEI by measuring the macronutrient composition of a menu and using the combustion enthalpy values of carbohydrates, proteins, and fats, to convert mass quantities to calories. For this work, we assume GEI values of 4.1 kcal/g for carbohydrates, 5.65 kcal/g for proteins, and 9.3 kcal/g for fats [21].

DEI is defined as the difference between GEI and the energy of waste materials leaving the digestive tract as fecal energy (FE) and combustible gas energy (GaE) (e.g., methane and H_2_) [22]:

DEI=GEI–FE–GaE (1)

Metabolizable energy is defined as the difference between GEI and all waste products produced by the body, including surface energy (SE) and urine energy (UE).


MEI=GEI–FE–GaE–UE–SE=DEI–UE–SE (2)

Surface energy includes skin shredding, perspiration, and hair loss. Atwater coefficients [23] often are used to estimate MEI: 4 kcal/g for carbohydrates, 4 kcal/g for proteins, and 9 kcal/g for fats.

As summarized in Table 1, we assume that, once absorbed, MEI for carbohydrates and fats are their GEI values. For proteins absorption, MEI is much lower than the GEI, because the body excretes reduced nitrogen in urine as urea, uric acid, creatinine, and creatine [21].

**Table 1:** GEI, Atwater coefficients, and post-absorption MEI for macronutrients in food.

	Carbohydrates	Proteins	Fats
Chemical Formula^a)^	CH_1.826_O_0.913_	CH_2.063_O_0.626_N_0.282_	CH_1.838_O_0.118_
Gross Energy (kcal/g)^a)^	4.1	5.65	9.3
Atwater Metabolizable Energy (kcal/g)	4	4	9
Post-Absorption Metabolizable Energy (kcal/g)	4.1	4.39^a)^	9.3

a) Ferrannini [21].

While caloric values are additive, weights (mass) are not additive. For example, adding 10 grams of proteins and 5 grams of fat does not make 15 grams of “fat plus protein.” A fundamentally correct and comparable unit for describing microbial reactions is the chemical oxygen demand (COD), which is a measure of the electron equivalents in the carbon of an organic compound [24]. Specifically, COD is the mass of oxygen (O_2_) required for the complete oxidation of an organic compound, based on the O_2_ half reaction:

$$O_{2}+4\;e^{-}+4\;H^{+}\to 2\;H_{2}O$$ (3)

where 1 mole of O_2_ (32 g of O_2_) accepts 4 mol of electrons, which provides the relationship 8 g COD = 1 electron equivalent (e^-^ eq). To define the COD of carbohydrates (CH_1.826_O_0.913_), proteins (CH_2.063_O_0.626_N_0.282_), and fats (CH_1.838_O_0.118_), we use the following reduction half reactions:

$$Carbohydrates:\;CO_{2}+4\;H^{+}+4\;e^{-}\to CH_{1.826}O_{0.913}+1.087\;H_{2}O$$ (4)

$$Proteins:\;CO_{2}+0.282\;NH_{4}{}^{+}+3.683\;H^{+}+3.965\;e^{-}\to CH_{2.063}O_{0.626}N_{0.282}+1.374\;H_{2}O$$ (5)

$$Fats:\;CO_{2}+5.602\;H^{+}+5.602\;e^{-}\to CH_{1.838}O_{0.118}+1.882\;H_{2}O$$ (6)

Because the formula weight of carbohydrates is 28.434 g and 4 e^-^ eq corresponds to 32 g COD, the COD value of carbohydrate is 32/28.434 = 1.125 g COD/g carb. Similarly, the COD values of proteins = 1.132 g COD/g prot, and fats = 2.850 g COD/g fat. The conversion from g COD to kcal for carbohydrates is 3.643 kcal/g COD, for proteins is 4.771 kcal/gCOD, and for fats is 3.263 kcal/g COD (Davis et al., 2020 [24]).

We use the following stoichiometry for determining the COD of the most common SCFAs:

$$Acetic\;Acid:\;C_{2}\;H_{4}O_{2}+2\;O_{2}\to 2\;CO_{2}+2\;H_{2}O$$ (7)

$$Propionic\;Acid:\;C_{3}H_{6}O_{2}+3.5\;O_{2}\to 3\;CO_{2}+3\;H_{2}O$$ (8)

$$Butyric\;Acid:\;C_{4}H_{8}O_{2}+5\;O_{2}\to 4\;CO_{2}+4\;H_{2}O$$ (9)

The COD value of acetic acid is 2(32)/60 = 1.066 g COD/g acetic acid, propionic acid is 3.5(32)/74 = 1.513 g COD/g propionic acid, and butyric acid is 5(32)/88 = 1.818 g COD/g butyric acid. The combustion enthalpy values of SCFAs 3.480 kcal/g acetic acid, 4.925 kcal/g propionic acid, and 5.1919 kcal/g propionic acid. Therefore, the gross energy values for acetic acid is 3.266 kcal/g COD, propionic acid is 3.258 kcal/g COD, and butyric acid is 3.260 kcal/g COD.

Because of its comparability and additivity based on electron equivalents, we use g COD to establish mass balance for the digestive tract. However, we also present our results in kcal to make them intuitive and relevant for practitioners in human nutrition and metabolism. We developed a method to convert between g COD and kcal in Davis et al. [24].

### 2.2 Model formulation for the upper GI

We formulated the model for the upper GI tract by making four broad assumptions:

1. In the small intestine, nutrient absorption is driven by the host’s physiological processes.
2. The upper GI tract adds various pancreatic secretions, gastric acids, bile acids, and mucus. Collectively, we refer to them as gastrointestinal secretions, GIS (kcal/d).
3. In the small intestine, most carbohydrates, proteins, and water-soluble vitamin absorption occur within the first 100 to 200 cm of the jejunum, while fat is absorbed through the length of the intestine [25, 26]. The remaining region that provides an excess capacity for absorption is termed the anatomical reserve. Thus, absorption in the small intestine is complete and remains fixed while the length of the small intestine is greater than its anatomical reserve length (ARL).
4. The efficiency of absorption in the small intestine decreases when surgery decreases the length of the small intestine below its ARL. We use percentages of the small intestine removed to avoid uncertainty in measuring the absolute length [27].

Based on these assumptions, we developed a two-part model for estimating the extent of dietary absorption in the small intestine and the content of the ileocecal output. The Excel document and a Python version with a complete implantation of the model can be found in the GitHub repository listed in Supplemental Material. We model the absorption of the upper GI, UGA (kcal/d), using a piecewise model that considers the length of the small intestine surgically removed (SIR) and the ARL (cm).

$$\begin{array}{l} UGA=GEI\left(\alpha -\beta \max(0,\frac{SIR-ARL}{SIL})\right)\\ \;=GEI(\alpha -\beta \max(0,\%\mathrm{Intestine}\;\mathrm{Removed}-\%\mathrm{Anatomical}\;\mathrm{Reserve}\;\mathrm{Length})) \end{array}$$ (10)

where SIL is the small-intestine length of a healthy person and α and β are model coefficients. The first term considers nutrient absorption with normal physiology, where α is the true digestibility of a macronutrient. The second term describes the inefficiency in macronutrient absorption caused by surgery; β describes the decrease in nutrient absorption when the length of small intestine removed, *SIR* (cm), exceeds the ARL (cm).

As shown in Fig 1, The ICO is the difference between GEI and the energy absorbed:

ICP=GEI−UGA+GIS (11)

It represents a combination of dietary substrates unabsorbed in the upper GI tract and the net secretions of the gastrointestinal tract that includes mucus, bile acids, pancreatic juices, and cell debris.

### 2.3 Digestibility coefficients for the upper GI model

Table 2 summarizes literature values for upper-GI digestibility coefficients and secretions of the gastrointestinal tract. These coefficients were estimated by measuring the contents of ileostomates (i.e., output from the terminal ileum for subjects who had their colon removed). While the processes in the upper GI tract are primarily absorptive, secretions of the gastrointestinal tract (e.g., bile acids, gastric acids, and pancreatic secretions) add carbohydrates, proteins, and fats into the upper GI. Studies estimate α (the true digestibility) for a dietary substrate by subtracting out the contribution of dietary secretions make to the ileostomy output:

**Table 2:** Survey of ileal digestibility coefficients from the literature and our estimates (this work).

**Digestibility Coefficient α (%)**			
Macronutrients	Diet	Value	Ref
Proteins	low nitrogen diet+whole soya beans	0.73	[16]
	low nitrogen diet+pureed soya beans	0.885	[16]
	high nitrogen diet	0.9	[16]
		0.947–0.953	[28]
		**0.9**	**This Work**
**Net Secretions of the GI Tract DS**			
Components	Diet	Value **(**gCOD/d**)**	Ref
Pancreatic enzymes and other gut secretions		4.5–6.8	[29]
Mucus		2.3–3.4	[30]
Proteins		5.7	This Work
Mucin Carbohydrates		3.4–4.5	[30]
Mucin Carbohydrates		3.2	[31]
Mucin Carbohydrates		**3.4**	**This work**
Fats		1.4 to 14	[32]
Fats		3.1	**This Work**


$$\alpha =1-\frac{\left(\mathrm{ICP}-\mathrm{GS}\right)}{\mathrm{GEI}}\times 100$$ (12)

For proteins, α ranges from 0.73 to 0.9, depending on the protein source. We use α = 90% as a general digestibility coefficient in the absence of specific dietary information. When additional information is available, for example that whole-plant-based protein is used, a lower digestibility coefficient may be used. We take 5.7 gCOD-protein/d as the secretion of total nitrogen, which agrees well with other estimates [29].

For fats, the net absorption coefficient ranges from 88% for a low-fat diet to 96% for a high-fat diet [32], because intake of a high-fat diet stimulates pancreaticobiliary secretions, which contain fat, and that can end up in the ileostomy effluent. Considering that fat absorption is generally efficient in the small intestine, we select α = 96% for this study and use fat secretion of 3.1 gCOD/d [32] from pancreatic and bile secretions.

For carbohydrates, we assume α = 90% for sugars. Approximately 3.2 to 4.5 gCOD/d of mucin carbohydrates are secreted in the intestines. Therefore, we use carbohydrates secretion of 3.4 gCOD/d. The α values for carbohydrates and fats are comparable to the values used by Remer et al. [33].

### 2.4 Model formulation for the lower GI

In the lower GI tract, microorganisms receive the contents of ICO as their substrates. The major source of substrates for microorganisms are carbohydrates in resistant starch and non-starch polysaccharides. Microorganisms consume polymeric substrates in two main steps. First, microorganisms hydrolyze the polymeric substrates into their monomeric constituents. Second, microorganisms ferment these monomeric growth substrates to mainly acetate, propionate, and butyrate in anaerobic conditions. We make four simplifying assumptions to formulate a model for microbe-driven digestion and absorption processes in the lower GI tract.

#### 2.4.1 Assumption 1

Hydrolysis is the limiting process for microorganisms fermenting carbohydrates and proteins.

Proteins and dietary carbohydrates (disaccharides, oligosaccharides, and polysaccharides) are polymeric and they must be hydrolyzed to their respective monomers before they are fermented by microorganisms [34]. We assume that the hydrolysis rate follows a first-order kinetics, the colon behaves as a single chemostat (i.e., a well-mixed reactor with continuous flow), and the overall rate of hydrolysis (*R*_hyd_, g COD/d) is:

$$R_{hyd}=M_{P}^{0}(\frac{1}{1+k_{hyd}\theta })$$ (13)

where M_P_^0^ are the mass flux (gCOD/d) of particulates in gCOD (carbohydrates and proteins) in the ileocecal output and feces, respectively; k_hyd_ is the hydrolysis coefficient (1/d); and *θ* is the colon’s transit time (d). Because the human colon has some plug-flow nature, the chemostat assumption tends to underestimate the hydrolysis rate and, presumably microbial metabolism; however, the effects on the overall trend is marginal [19].

#### 2.4.2 Assumption 2

The following are typical molar ratios for the common SCFAs: 1 Ac: 0.31 Prop: 0.15 n-But [35] in human fecal samples, although they can vary. For simplicity, we assume this ratio as the average of colon’s microorganisms and fix the reaction stoichiometry for carbohydrates (Eq 14) and proteins (Eq 15):

$$CH_{1.826}O_{0.913}+0.042\;NH_{3}\to +0.042\;C_{5}H_{7}O_{2}N+0.206\;C_{2}H_{4}O_{2(Acetic\;Acid)}+0.064\;C_{3}H_{6}O_{2(Propionic\;Acid)}+0.031C_{4}H_{8}O_{2(n-Butyric\;Acid)}+0.063\;CO_{2}+0.102\;H_{2}O$$ (14)

$$CH_{2.063}O_{0.626}N_{0.282}+0.180\;H_{2}O\to 0.020\;C_{5}H_{7}O_{2}N+0.261\;NH_{3}+0.199\;C_{2}H_{4}O_{2(Acetic\;Acid)}+0.043\;C_{3}H_{6}O_{2(Propionic\;Acid)}+0.043\;C_{4}H_{8}O_{2(iso-Butyric\;Acid)}+0.016\;C_{7.25}H_{7.2}O_{1.2}N_{0.2(Aromatics)}+0.091\;CO_{2}$$ (15)

In the reaction stoichiometry, microbial biomass is C_5_H_7_O_2_N [36]; no energy-embedding gases (H_2_ and CH_4_) are generated. The fermentation stoichiometries for carbohydrates and proteins differ in two ways. First, carbohydrate fermentation produces SCFAs like n-butyric acid, while protein fermentation produces branched chain fatty acids like iso-butyric acid. Second, a signature for protein degradation is the generation of aromatics, which typically occurs in the distal regions of the colon. In particular, microbiological degradation of amino acids with aromatic functional groups produces aromatic degradation products such as phenylacetate and phenylpropionate from phenylalanine; cresol, phenylacetate, and phenylpropionate from tyrosine; and indole acetate or indole propionate from tryptophan [37]. We use the formula C_7.25_H_7.2_O_1.2_N_0.2_ to represent these aromatic products.

#### 2.4.3 Assumption 3

Most SCFAs (95%) generated in the colon are absorbed, and 5% leave the colon with the feces [38–40].

#### 2.4.4 Assumption 4

In the lower GI tract, we also consider a small amount of fat absorption in the colon using:

$$\frac{M_{fat}}{M_{fat}^{0}}=\frac{1}{1+k_{abs}\theta }$$ (16)

where *k*_abs_ is the absorption rate of fat by the colon. Solving for k_abs_ gives:

$$k_{abs}=(1+\frac{M_{fat}}{M_{fat}^{0}}){\left(\theta \frac{M_{fat}}{M_{fat}^{0}}\right)}^{-1}$$ (17)

### 2.5 Model input

We used data from Nordgaard et al. [15] for parameter fitting for the upper GI and for model evaluation for the overall GI tract. Nordgaard et al. [15] performed fecal measurements on 148 human subjects (92 women, 56 men) with varying lengths of the colon and small intestine removed. On average, subjects consumed 2370 ± 50 kcal/d (90% CI: 1900–2600 kcal/d). The macronutrient composition was 55% carbohydrates (CI: 46%-57%), 22% proteins (CI: 20%-24%), and 32% fat (CI: 29%-36%). The reported energy amount and macronutrient composition had large standard deviations, because the food items were not standardized, but tailormade based on a dietary questionnaire to satisfy the needs of each subject. This means that establishing an exact mass balance is not possible, but we can analyze the trends from the mean values for all subjects. We assume dietary input of 2370 kcal per day with the macronutrient composition of 50% carbohydrates, 20% proteins, and 30% fats.

Table 3 summarizes g COD and kcal values for the model input. Metabolizable energy was calculated by assuming the Atwater coefficients of 4 kcal/g for carbohydrates, 4 kcal/g for proteins, and 9 kcal/d for fats. We subcategorized carbohydrates into Available Starch and Sugars, Resistant Starch (RS), and Non-Starch Polysaccharides (NSP). We assumed that RS and NSP constitute dietary fibers. Nordgaard et al. [15] reported average dietary fiber of 22.9 ± 0.6 g/d. According to Cummings and Macfarlane [29], a Western diet consists of 8 to 18 g/d of NSP and 8 to 40 g/d of RS. In the absence of specific information, we equally proportionated dietary fibers into NSP and RS.

**Table 3:** Summary of dietary intake as grams/d, gCOD/d, and kcal/d.

Nutrients	Amount (g/d)	Formula	COD	GEI	MEI
			(gCOD/d)	(kcal/g)	(kcal/d)
Protein	83.9	CH_2.063_O_0.626_N_0.282_	94.9	474	336
Fat	76.5	CH_1.838_O_0.118_	217.9	711	688
Carbohydrates					
Available Starch & Sugars	266.1	CH_1.826_O_0.913_	299.5	1091	1064
Resistant Starch (RS)	11.5		12.9	47	46
NSP	11.5		12.9	47	46
Total	452		638.1	2370	2180

Coefficients for Gross Energies are 4.1 kcal/g for carbohydrates, 5.65 kcal/g for proteins, and 9.3 kcal/g for fat [21, 24]. Metabolizable energies are 4 kcal/g for carbohydrates, 4 kcal/g for proteins, and 9 kcal/g for fat. Abbreviations are COD—Chemical Oxygen Demand; GEI–Gross Energy Intake; and MEI–Metabolic Energy Intake.

### 2.6 Parameter fitting

Model parameters were calculated using the least square (χ^2^) regression of the data, which is defined as sum of the squared difference between the measurement (y) and model prediction (y*) for a dependent variable

$$\chi^{2}=\sum_{i}{\left(y_{i}-y_{i}^{*}\right)}^{2}$$ (18)

where the subscript *i* describes one datapoint. For the upper GI tract, the parameters that were fitted are α, β, and %ARL, which were fit using the measurements of the ICO for the patient group that had 100% of the colon removed and variable amounts of the small intestine removed [15]. For the lower GI tract, the fitted parameters were the hydrolysis coefficients of resistant starch (*k*_hyd,carb_) and proteins (*k*_hyd,carb_) and the absorption rate of fat (*k*_abs_). These parameters were obtained by considering the measured fecal contents of proteins, fats, and carbohydrates for the patient group that has had 100% of the colon intact and variable amounts of the small intestine removed [15].

For describing the proportion of variance explained by independent variables using our model, we calculated the coefficient of determination as

$$R^{2}=1-\frac{SS_{res}}{SS_{tot}}=\frac{\sum_{i}{\left(y_{i}-f_{i}\right)}^{2}}{\sum_{i}{\left(y_{i}-\bar{y}\right)}^{2}}$$ (19)

where *SS*_*res*_ is the residual sum of squares, *SS*_*tot*_ is the total sum of squares, *y*_i_ are individual observation, $\bar{y}$ is the average of all observations, and *f*_*i*_ is the model prediction.

## 3. Results and discussion

### 3.1 Parameter fitting for the upper GI model

Nordgaard et al. [15] measured carbohydrates, fats, and proteins in the feces for patients who had portions of the intestines removed. Here, we consider two subgroups as shown in S1 Fig: Group A (n = 66) contained subjects who had undergone total colonic resection. We used Group A to estimate the composition of the ICO. Group B (n = 33) contained subjects who had the full colon intact, but with varying amounts of small intestine removed (0 to 95%). We used Group B for estimating the impact of removing the small intestine on the overall energy uptake. We used data from Groups A and B to evaluate the ability of our model to quantify separate contributions that physiological processes (in the upper GI tract) and microbiological processes (i.e., SCFAs production in the colon) made to overall energy absorption.

**Fig 2:**
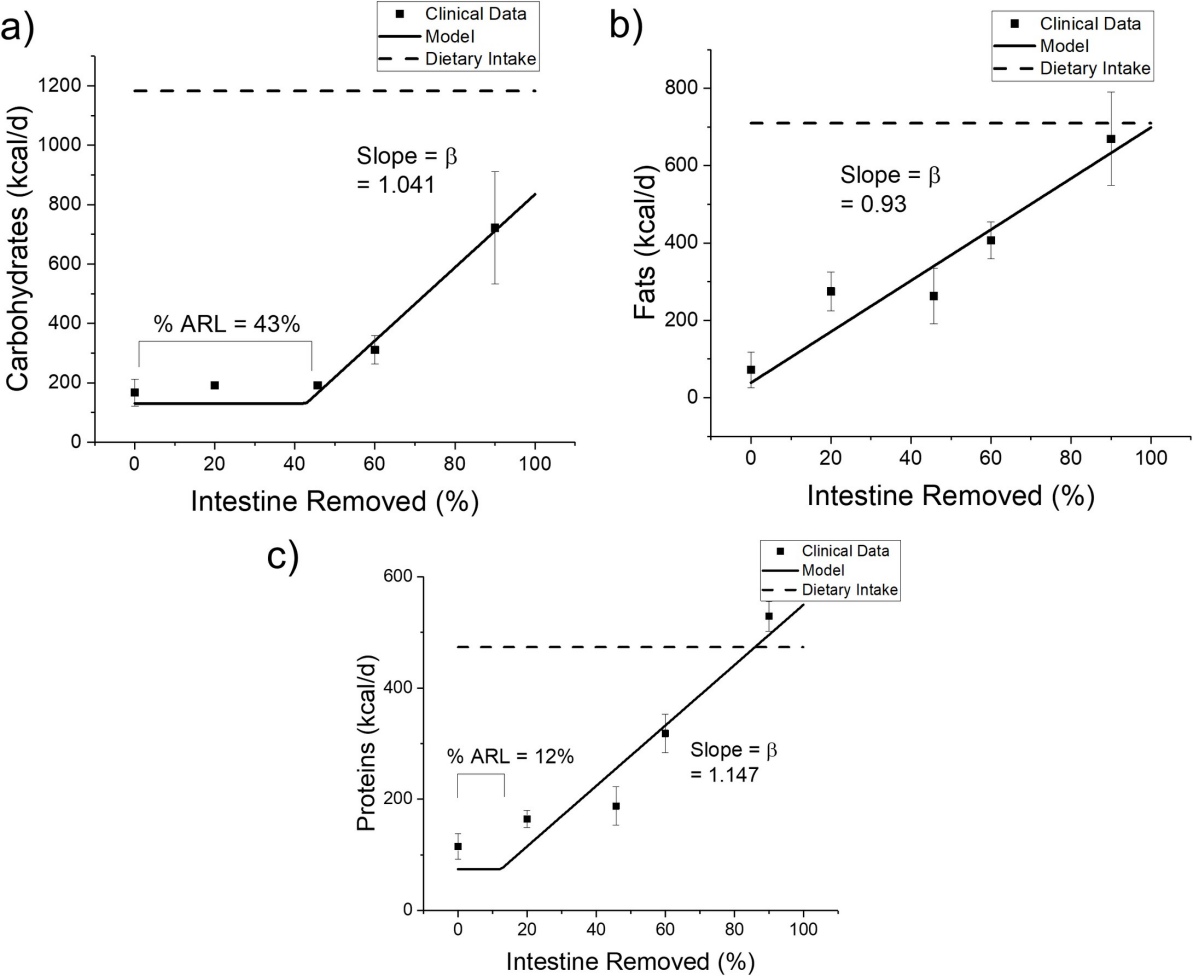
The effect of varying degrees of small-intestine resection on Ileocecal output (ICO) of carbohydrates, fats, and proteins. Model estimates using Eq 10 (solid lines) agree well with a dataset (points) from Nordgaard et al. [15]. Each data point represents an average of the measurements on multiple subjects (n = 2 to 15) with the error bar describing the standard error. Dietary intake is shown as dotted lines. % ARL stands for the percentage of anatomical reserve length as defined in Eq 10. The coefficient of determination for carbohydrate R^2^ = 0.9698, fat R^2^ = 0.8954, and protein R^2^ = 0.9085.

We fit Eqs. 1 and 2 to the clinical data for Group A by minimizing the least squares to parameterize the model for the upper GI. Fig 2 shows the data and model fits, and Table 4 summarizes our estimates of β and %ARL. Fig 2 shows how the energy content of carbohydrates, proteins, and fat in the dietary intake (dotted line) and ileocecal output (circle = clinical data; solid line = our model) varied with the percentage of small intestine removed. The model captures well the trends in the clinical data. On the one hand, the amount of fats in the ICO increased in proportion to the percent of small intestine removed. On the other hand, carbohydrate and protein contents of the ileostomy effluent changed little when only a small segment of the small intestine was removed. The best %ARL values are estimated to be 43% for carbohydrates, 11% for proteins, and 0% for fats.

**Table 4:** Digestibility parameters for macronutrients for the upper GI.

	Units	Carbohydrate	Protein	Fat
Α		0.90	0.90	0.96
β		1.041	1.147	0.930
%ARL		43%	11%	0%
DS	kcal/d	12.3	27.0	10.2

Abbreviations and symbols are α—the upper GI absorption coefficient for normal physiology; β—a coefficient for inefficiencies in upper GI absorption due to surgery; % ARL—the percentage of anatomical reserve length as defined in Eq 11; and GIS–gastrointestinal secretions.

Our model indicates that, if 100% of the small intestine were removed, the upper GI tract still would absorb 29% of carbohydrates and 2% of fats, but its net addition of protein would be 16%, from secretions. Because the clinical data only extend up to 92% and these model outputs extend beyond the range of regression, we recognize limits to interpreting the results. However, the model results suggest which mechanisms are at work. The modest absorption of carbohydrates may indicate the presence of some microbiological growth or physiological adaptation in the small intestine. The distal region of the remaining small intestine can contain microorganisms that consume carbohydrates, which are comparatively more biodegradable. After a large fraction of the small intestine, a process known as intestinal adaptation occurs whereby the remaining small intestine (mostly the ileum and maybe proximal colon to some degree) undergoes a variety of structural and functional changes in order to try to improve its absorptive capacity. For example, the small intestine can adapt physiologically by increasing the expression of transporters for sugars and amino acids for enhanced physiological uptake or slowing the small intestinal transit time to increase contact time for nutrient absorption [41]. According to Table 2, gastrointestinal secretions, such as pancreatic acids and mucus and cell debris, contribute about 4 to 6 gCOD/d of protein loading (or about 6% of dietary intake), which was lumped into the gross measurements of protein made by Nordgaard et al. [15]. In healthy subjects, gastrointestinal secretions are reabsorbed in the small intestine; however, it is possible that these secretions were poorly absorbed in individuals with small intestine resection and were not degraded by microorganisms, if there were any.

### 3.2 Effects of small intestine resection on fecal energy

**Fig 3:**
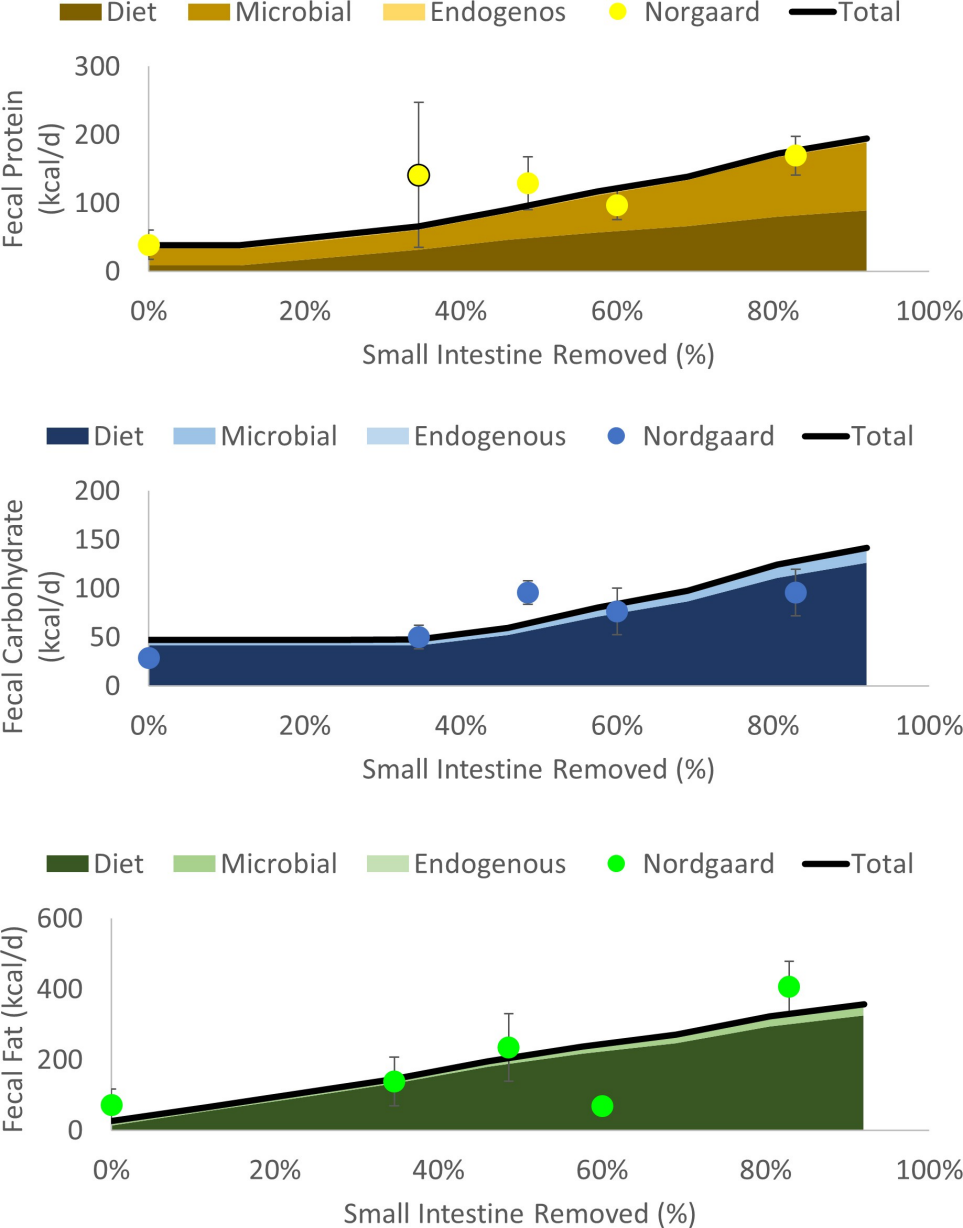
The effect of varying degrees of small-intestine resection on fecal content leaving the colon after digestion by the colonic microbiota. Model estimates of dietary, microbial, and endogenous secretions of macronutrients are shown as shaded areas, and their total are shown as the solid lines. The clinical data from Nordgaard et al. [15] are shown as points.

Fig 3 and S2 Fig compare the fecal output of macronutrients for our model and the clinical data from Nordgaard et al. [15] for subjects with 100% of the colon intact, but with varying degrees of the small intestine removed. We obtained the following model parameters separately by minimizing the least square errors (Eq 8) and considering the total contents of proteins in feces, carbohydrates, and fats: *k*_hyd,RS_ = 2.8 d^-1^, *k*_hyd,protein_ = 2.2 d^-1^, and *k*_abs_ = 0.48 d^-1^. The parameters for carbohydrates and fats were optimized before proteins, because microbial growth on carbohydrates have downstream effects on the protein contents. The hydrolysis coefficients obtained for proteins and RS fall near the upper end of hydrolysis coefficients typically observed in microbiological systems [34].

For all macronutrients, the model captures three overall trends. First, energy measured in feces increased as a higher percentage of the small intestine was removed. Second, microbial output (in feces) was greater than diet output for proteins in all cases, while diet output was dominant for carbohydrates and fats. Microbe-derived output was much more important for protein in part because microbial biomass is about 55% protein. The clinical data for proteins show an odd increase at 35% small intestine removed, which has a wide error bar that may be a result of the non-standardized diets consumed among participants. Finally, contributions from endogenous sources were small in all cases.

**Fig 4:**
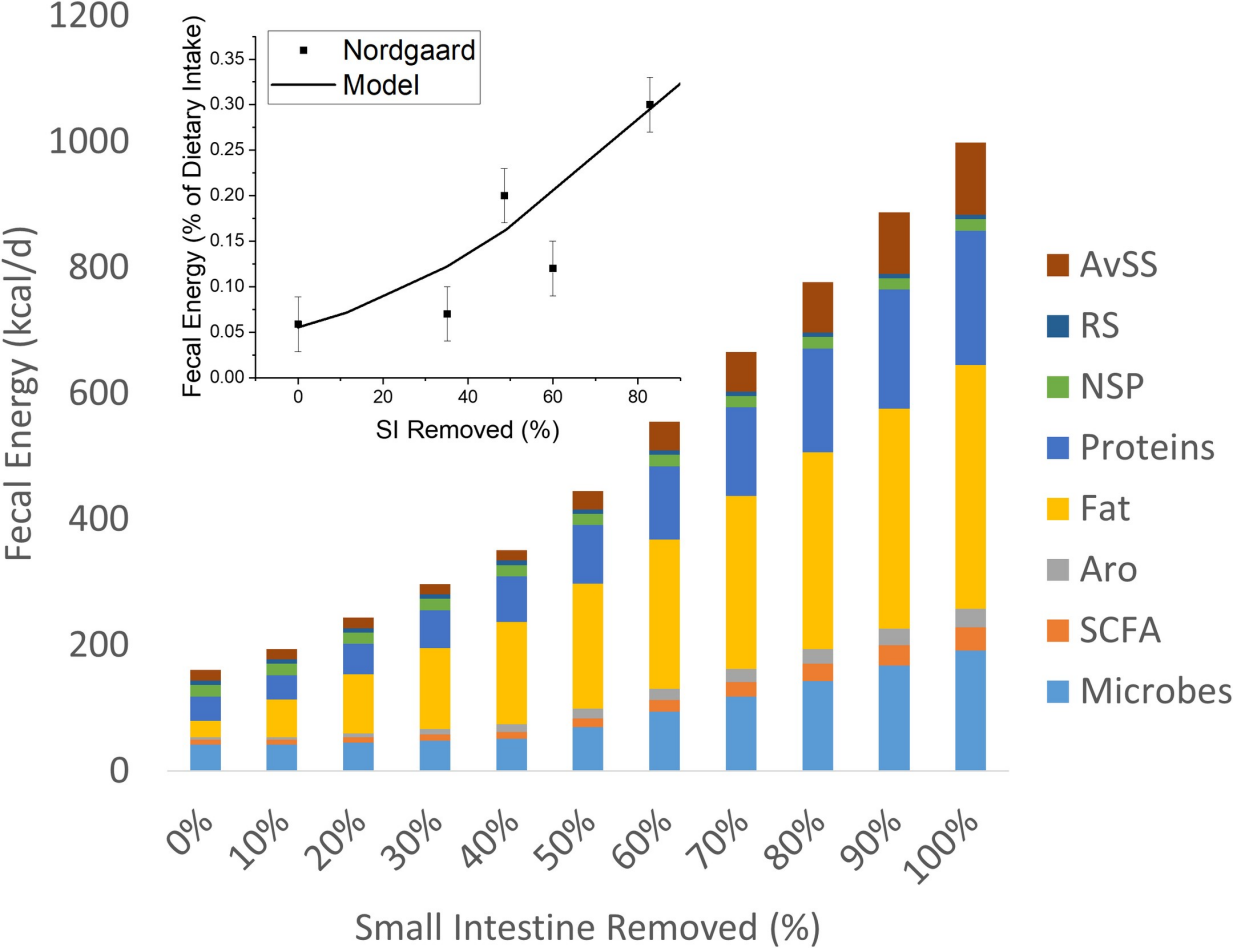
The effects of removing a percentage of the small intestine (x-axis) on the stool composition (y-axis). Fats, proteins, and carbohydrates here are composites of dietary and secretary sources. While microbial biomass contains these macronutrients, the model tracks them separately as microbial biomass. The inset compares the total fecal energy for our model and clinical data from Nordgaard et al. [15]. Abbreviations are AvSS–Available starch and sugars; RS–resistant starch; NSP–non-starch polysaccharides, Aro–aromatics; and SCFA–short chain fatty acids.

Fig 4 shows good agreement between the total fecal calories predicted by our model and clinical results of Nordgaard et al. [15]. As the percentage of the small intestine removed increased, the fecal output increased. Under a near complete small intestine resection, the fecal energy increased more than 3-fold compared to normal physiology. The inset in Fig 4 shows that fat energy measured in feces had the greatest sensitivity to removal of the small intestine, rising to 50% of the total energy output when the small intestine was 100% removed. Microbial biomass contribution to total fecal energy also become more important above about 50% of the small intestine removed.

### 3.3 Effects of small intestine resection on nutrient absorption

**Fig 5:**
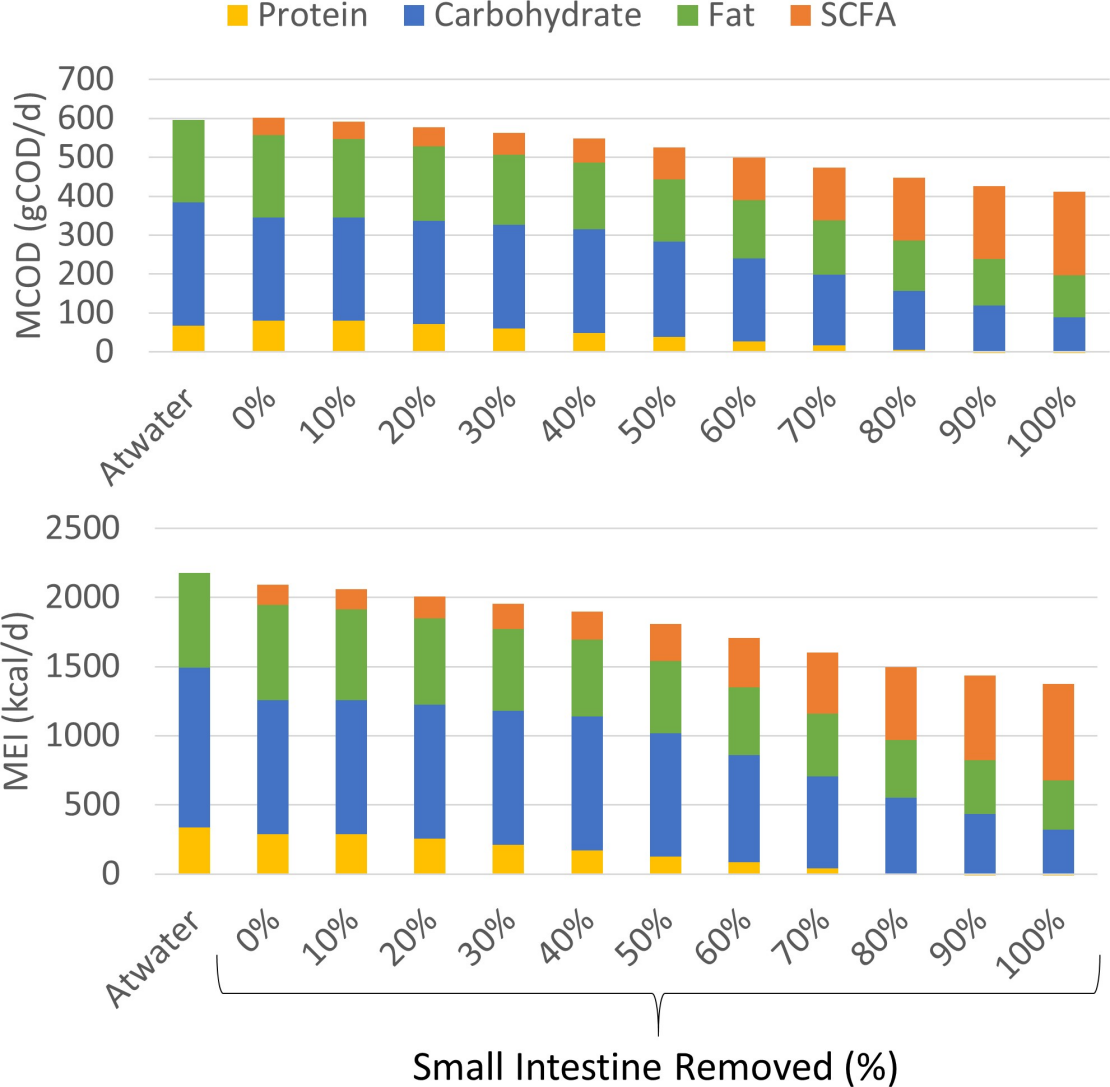
The effects of small intestine resection on the composition of a) COD and b) energy absorbed by the body. The calculations for the Atwater coefficients use typical metabolizable energy values listed in Table 1 and represents normal physiology.

Fig 5 shows the modeled effects of removing a percentage of the small intestine on the composition and amount of macronutrient absorbed by the small + large intestines, represented as metabolizable COD (top panel; MCOD) and metabolizable energy (bottom panel; MEI). Our model indicates that a person with a fully intact small intestine absorbs 2093 kcal/d of MEI and 602 g COD/d of MCOD. In comparison, the Atwater coefficients predict absorption of 2180 kcal/d of MEI and 595 g COD/d of MCOD. Because SCFAs derived from carbohydrates and proteins have lower energy values than the original substrates, the model prediction shows a lower energy absorbed than the Atwater despite having comparable COD values. The “lost” energy value is harvested by the microbes during fermentation.

As the small intestine is progressively removed, energy absorbed by physiological processes in the upper GI decreases, and, in parallel, the energy absorbed by the colon increases, while fecal energy increases. Thus, uptake of microbially produced SCFAs in colon becomes the largest source of energy uptake for the host as the loss of the small intestine approaches 100%. This shift to SCFA uptake in the colon partially compensates for the loss of uptake in the small intestine. For example, even with 100% of the small intestine removed, the combination of the small intestine and the colon recovers a substantial fraction of the dietary COD intake, 62% compared to 92% for an intact small intestine. Thus, the model reinforces the critical role of the colon in recovering energy as the upper GI tract is compromised [15]. In particular, when the percentage of the small intestine removed exceeds that of the anatomical reserve length (>45%), dietary absorption in the upper GI tract decreases, and SCFAs increasingly become an important source of energy for the body. The shift in energy absorption from macronutrients (fats, proteins, and carbohydrates) to SCFAs may have important roles in the health and recovery of patients who have undergone resection of the small intestine.

This study did not consider the effects of microbiome composition since the data were collected by Norgaard et al. [15] before the advent of high-throughput sequencing technologies. Instead, we systematically considered the dietary intake, small intestine absorption, and small intestine resection to estimate the ileocecal output, which is the input for the microbiome model representing the food available to microorganisms. Because an input boundary condition is necessary for mathematical modeling of the colon’s microbiome, we believe our method of estimating the ileocecal output will be complementary to existing mathematical models for the colon’s microbiome [42]. While we kept our representation of the microbial community purposefully simple, the combination of laboratory experiments and clinical studies should provide opportunities to refine the representation of the colon’s microbial community [18, 42].

## 4. Conclusion

We developed a mathematical model to provide an estimate of ICO, the nutrients made available to microorganisms in the colon, for humans with normal physiology and those who had sections of the small intestine removed. This makes it possible to quantify how surgical interventions such as small intestinal resection affect the microbiological activity in the colon. It also helps contextualize the amount and form of energy microorganisms recover for the human body. It is well known that under normal physiology conditions, SCFAs derived from microorganisms provide the human host with 5–10% of daily energy uptake. When energy absorption in the upper GI tract becomes inefficient due to the resection, microorganisms in the colon compensate by converting carbohydrates and proteins in the ICO to SCFAs. When a large portion of the small intestine is removed, SCFAs derived from microorganisms can become the dominant energy source for the body, not the intake food. These conversions enable us to study the effects of human physiology and surgical intervention on the colonic microorganisms, which is an important first step in quantifying the contributions that microorganisms make to the human energetics under clinically relevant conditions. A fruitful next step is developing a human bioenergetics model that quantifies the effects of SCFAs on the host’s metabolism.

We currently have an ongoing study in which participants are placed in two controlled diets for 22 days each and are in a metabolic ward for 11 days under energy-balance conditions [43]. We monitor the energy fluxes of participants through indirect calorimetry and continuous measurements of feces, urine, and gaseous products. We anticipate that our state-of-the-art clinical study will provide additional data for evaluating our model and opportunity for deepening the contributions that the microbiome makes to human energy expenditure by interfacing with human bioenergetics models [44, 45]. The current work will provide an important framework for this new analysis.

## List of abbreviations: (Alphabetical)

**Table:** 

**Acronym**	**Units**	**Name**	**Definition**
ARL	cm	Anatomical reserve length	The length of distal regions of small length that does not contribute to nutrient absorption
AvSS	Not applicable	Available starch and sugar	sum of available starch and sugars
COD	gCOD · g^-1^	Chemical oxygen demand	Measure of the mass of oxygen required for complete oxidation of the organic carbon in the sample
DEI	kcal · d^-1^	Digestible energy intake	The rate difference between GEI and the energy of waste materials leaving the digestive tract as stool
FE	kcal · d^-1^	Fecal energy	The energy rate of feces
GaE	kcal · d^-1^	Combustible gas energy	The caloric rate of combustible gases (i.e., methane and hydrogen) released by the body
GEI	kcal · d^-1^	Gross energy intake	The caloric rate of daily food intake
GI	Not applicable	Gastrointestinal	Abbreviation for gastrointestinal
GIS	kcal · d^-1^	Gastrointestinal secretions	Secretions rate of the gastrointestinal tract
ICO	kcal · d^-1^	Ileocecal output	The caloric rate of digestive materials entering the colon that includes undigested foods and gastrointestinal secretions
*k*_hyd_	d^-1^	Hydrolysis coefficient	The first-order hydrolysis-rate coefficient for dietary substrates
LGA	kcal · d^-1^	Lower GI absorption	The absorption rate of nutrients in the lower GI tract
MCOD	gCOD · g^-1^	Metabolizable COD	The difference between gross COD rate of food intake and the COD rate of waste materials leaving the digestive tract as stool and leaving the body as urine
MEI	kcal · d^-1^	Metabolizable energy intake	The rate difference between GEI and the energy of waste materials leaving the digestive tract as stool, urine, and surface energy
M_P_^0^	gCOD · d^-1^	Mass flux of particulate substrates	The mass flux of particulate substrates in the ileocecal output and stool
NSP	Not applicable	Non-starch polysaccharides	Polysaccharides in diet that includes lignin, cellulose, and hemicellulose
RS	Not applicable	Resistant starch	Resistant starch
SCFA	Not applicable	Short Chain Fatty Acids	A collection of small organic acids produced by microbes that includes acetic acids, propionic acid, n-butyric acid, and iso-butyric acid
SE	kcal · d^-1^	Surface energy	Energy rate carried by shedding of skin and hair and perspiration
SIR	cm	Small intestine surgically removed	The length of the small intestine surgically removed
SIL	cm	Small intestine length of a healthy person	The anatomical length of the small intestine
*θ*	d	Colon’s transit time	The transit time of digestive material through the lower GI
UE	kcal · d^-1^	Urine’s energy	The energy rate of urine
UGA	kcal · d^-1^	Upper GI absorption	The rate of absorption of nutrients in the upper GI

## Supporting information

S1 Fig Participant flowchart. (PDF)

S2 Fig Fecal COD trends. (PDF)

S1 File The link to a GitHub link to the Excel version and the Python version for this study. (DOCX)
