# Supplementary material for: Developing a model for estimating the activity of colonic microbes after intestinal surgeries
Source: PLoS One. 2021 Jul 28;16(7):e0253542. doi: 10.1371/journal.pone.0253542 (PMC8318292; doi:10.1371/journal.pone.0253542)
Supplement: S1 Fig — (PDF) [file pone.0253542.s001.pdf]

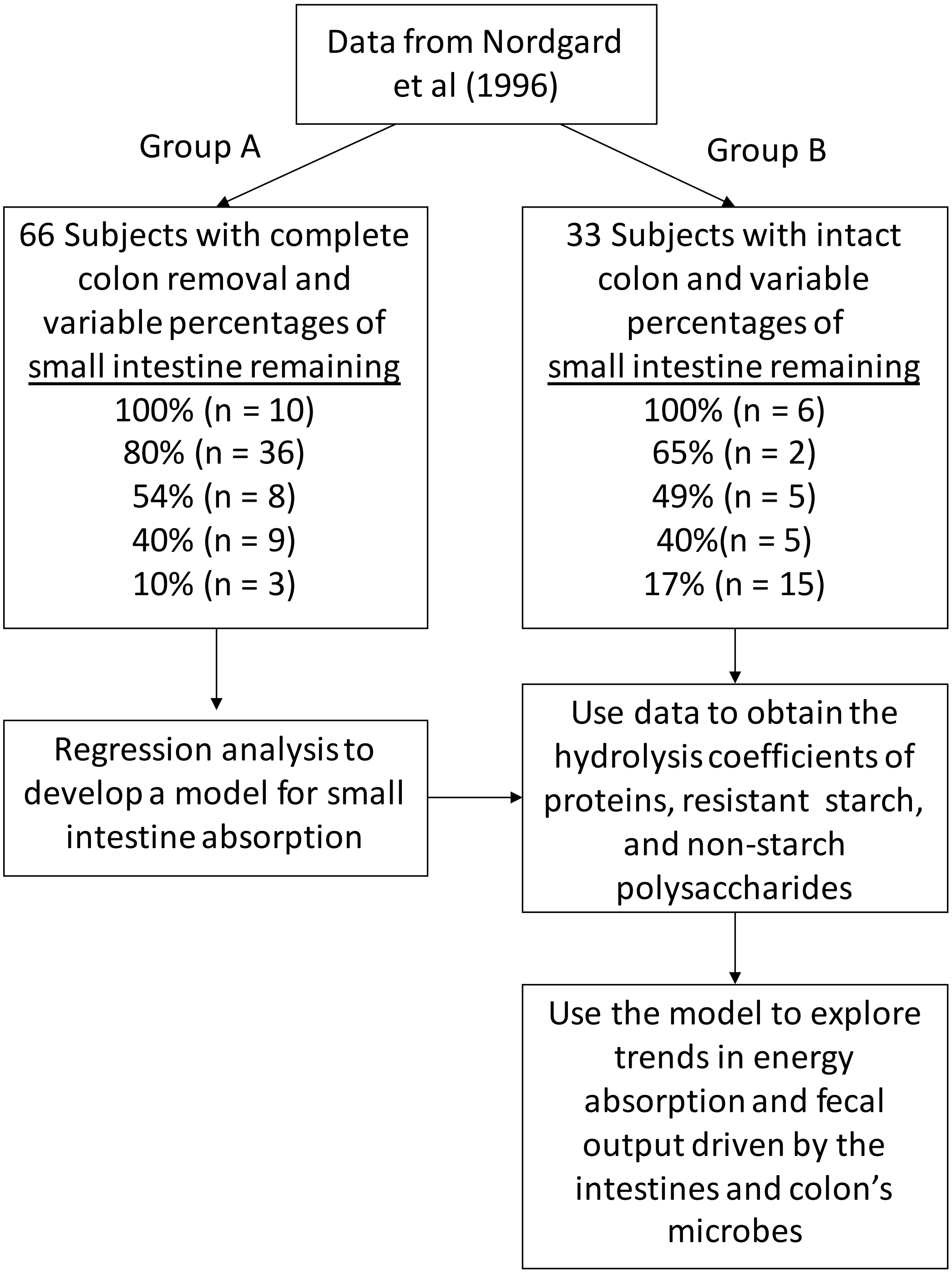

Figure S1. The participant flowchart describing how we constructed our model and used it for exploring the trends. The original data was collected by Nordgaard et al. (1) in 1996 before clinicaltrials.gov was established. They divided subjects into groups based on how much of the colon and small intestine are remaining. The lengths of the small intestine remaining were listed as >350 cm, 200-350 cm, 150-200 cm, 100-150 cm, and < 100 cm. We converted these lengths to the percentages of small intestine remaining by reading the averaged lengths of the small intestine for each patient group as listed in Figure 4 of Nordgaard et al. (1) and dividing them by 350 cm.
