# Supplementary material for: Developing a model for estimating the activity of colonic microbes after intestinal surgeries
Source: PLoS One. 2021 Jul 28;16(7):e0253542. doi: 10.1371/journal.pone.0253542 (PMC8318292; doi:10.1371/journal.pone.0253542)
Supplement: S2 Fig — (PDF) [file pone.0253542.s002.pdf]

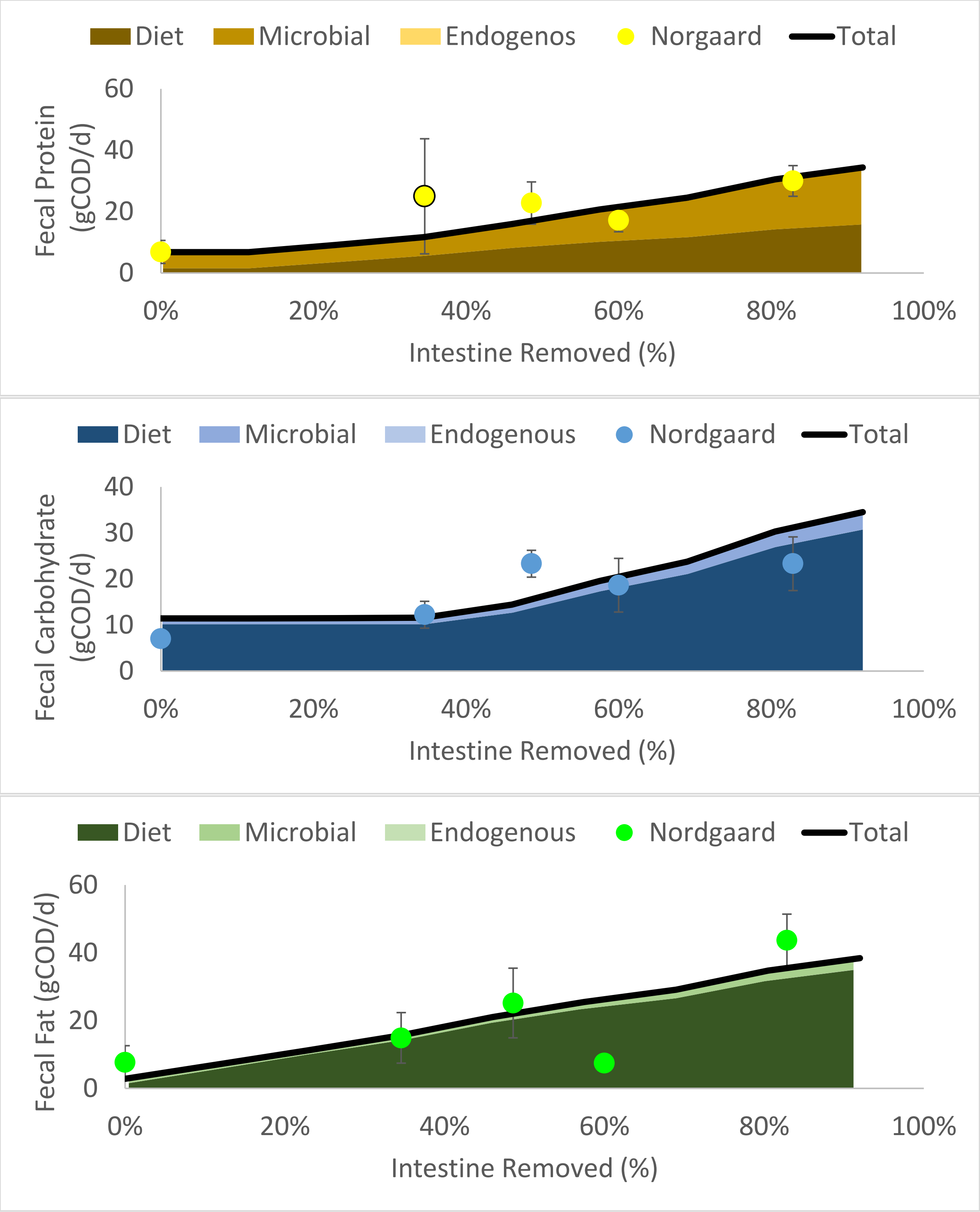

Figure S2. The effect of varying degrees of small-intestine resection on fecal content leaving the colon after digestion by the colonic microbiota. Model estimates of dietary, microbial, and endogenous secretions of macronutrients are shown as shaded areas, and their total are shown as the solid lines. The clinical data from Nordgaard et al. (1) are shown as points.

References

1. Nordgaard I, Hansen BS, Mortensen PB. Importance of colonic support for energy absorption as small-bowel failure proceeds. Am J Clin Nutr. 1996 Aug;64(2):222–31.
