# Supplementary material for: Developing a model for estimating the activity of colonic microbes after intestinal surgeries
Source: PLoS One. 2021 Jul 28;16(7):e0253542. doi: 10.1371/journal.pone.0253542 (PMC8318292; doi:10.1371/journal.pone.0253542)
Supplement: S1 File — (DOCX) [file pone.0253542.s003.docx]

**Online GitHub supplementary programs/spreadsheets**

The Excel document and the Python version used to calculate the overall and microbial contributions to digestivity and metabolic energy is in a public GitHub Repository. It can be accessed at the following link: <https://github.com/amarcus1/Metabolizable-and-digestible-energy-calculator-for-patients-with-small-intestine-removed>. The Calculator allows users to change the percentage of small intestine surgically removed in Parameters sheet.
